# Metastasizing Pleomorphic Adenoma: A Fascinating Enigma

**DOI:** 10.1155/2012/148103

**Published:** 2012-10-16

**Authors:** Luis E. IV Santaliz-Ruiz, Gary Morales, Héctor Santini, María Sánchez-Santiago, Axel Arroyo

**Affiliations:** ^1^Division of Otolaryngology Head and Neck Surgery, Department of Surgery, Damas Hospital, Ponce School of Medicine Consortium, Ponce, PR 00717, USA; ^2^Department of Cytotechnology, University of Puerto Rico, Medical Sciences Campus, San Juan, PR 00936, USA

## Abstract

Among salivary gland neoplasms, metastasizing pleomorphic adenoma (MPA) constitutes an extremely rare group of tumors. The present paper reports a case of pleomorphic adenoma (PA) in submandibular gland that, after more than 30 years of initial treatment, recurred and metastasized to ipsilateral neck lymph nodes and parotid gland. In an attempt to elucidate the malignant behavior of metastasizing pleomorphic adenoma, we performed Ki-67, p53, p16, and bcl-2 immunohistochemistry staining of our case sample. Many immunohistochemistry staining studies have been done on malignant salivary gland tumors. However, to the best of our knowledge no immunohistochemistry staining of the aforementioned markers has been previously performed on metastasizing pleomorphic adenoma.

## 1. Introduction

Salivary gland tumors represent 1% to 4% of all human neoplasias [1]. Pleomorphic adenoma (PA) is the most common tumor that affects the aforementioned glands [1–3]. Among salivary gland neoplasms, metastasizing pleomorphic adenoma (MPA) constitutes an extremely rare group of tumors [2, 3]. The World Health Organization (WHO) defines MPA as a “histologically benign pleomorphic adenoma that inexplicably manifests local or distant metastasis” [3]. Although apparently benign, the MPA-associated mortality could be as high as 22% [2]. The most common initial tumor site in MPA is the parotid gland (74%), followed by minor salivary glands (17%) and submandibular glands (10%). Most patients (81%) with MPA have a history of at least 1 local recurrence of PA prior to the detection of distant metastasis. In only 17% of patients the detection of MPA resulted from the medical evaluation of a local recurrent PA. The most common site for metastasis was the bone (45%), followed by the head and neck (43%), lungs (36%), and abdominal viscera (10%). Within the head and neck area, only 17% of cases metastasized to regional lymph nodes. On average, MPA usually occurs 16 years after the treatment of the initial benign PA [3]. The present paper reports a case of PA originated in the submandibular gland that eventually, after more than 30 years of initial treatment, recurred and metastasized to ipsilateral neck lymph nodes and parotid gland. 

Several hypotheses have been formulated to explain how a histological benign tumor may produce metastasis, such as previous surgical intervention of a primary PA that should favor permeation of blood vessels or lymphatic vessels by tumor cells, followed by metastatic spread [2]. However, the data in literature have shown that they are not absolute requisites for metastatic disease to occur [2, 4, 5]. The paradoxical nature of MPA, with its benign histological appearance and its metastatic spread capacity, intrigues clinicians and academic scholars.

## 2. Case Presentation

**Figure 1:**
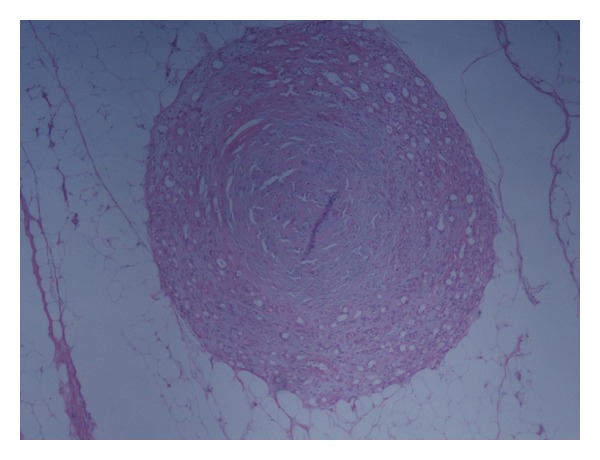
Well-delimited nodular lesion with a predominant center of myxoid changes and surrounding areas of epithelial components in 58 out of 59 neck nodules positive for MPA.

**Figure 2:**
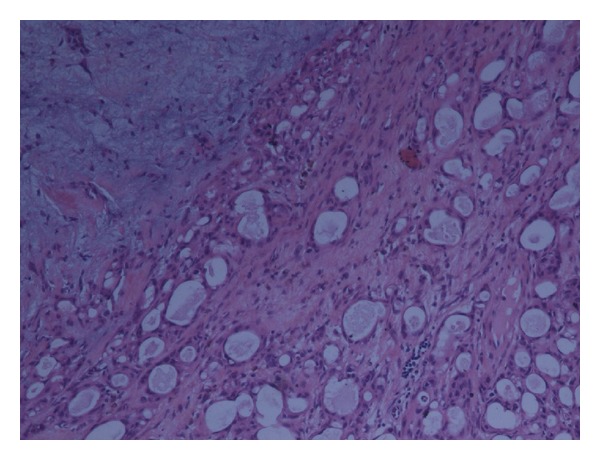
Predominant epithelial tubular component surrounded by myxoid components in salivary gland lesion.

**Figure 3:**
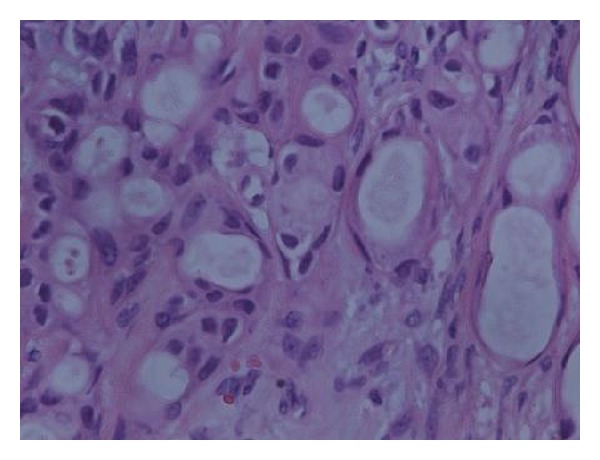
High power inset showing the epithelial component of tubular morphology with regular nuclei without atypia.

A 53-year-old female presented with complaint of soft tissue swelling on the left side of neck that had gradually increased in size over a 1-year period. Her past medical history is remarkable for left submandibular gland PA treated with enucleation more than 30 years ago. The details of the operative report from this first procedure were not available. On physical examination, head and neck revealed a 4 × 3.5 × 3 cm well-defined-enlarged-multilobulated submandibular gland. No pain or signs of facial nerve weakness or palsy were present. MRI of orbit, face, and neck without IV contrast showed ipsilateral cervical adenopathies and fluid signal intensity lymph nodes. The largest node measured 1.2 × 1.1 cm. Chest X-ray was unremarkable. Fine Needle Aspiration Biopsy of left submandibular gland yielded myoepithelial cells and myxoid stroma consistent with pleomorphic adenoma. Selective Left Neck Dissection of IA, IB, IIA, IIB, and III levels and total left parotidectomy were performed. Facial nerve and all its branches were identified and preserved during surgery with the aid of a nerve monitor. No tumor involvement of facial nerve was found. Despite nerve monitoring and appropriate nerve preservation, the patient shows a residual left facial nerve marginal branch weakness. Intraoperative pathology consultation revealed left submandibular gland with characteristic epithelial proliferation and focal myxoid changes of pleomorphic adenoma. Permanent histopathology diagnosed MPA of the left submandibular with 58/59 nodules positive for the lesion as well as 3 out of 5 left parotid gland nodules positive for lesion. The margins of surgical specimen were free of tumor (see Figures 1, 2, and 3). Therefore, this patient presented with local recurrence and simultaneous metastatic disease. Immunohistochemistry staining done on this tumor tissue revealed positive immunoreactivity for p16 (similar positive immunoreactivity in both nuclear and cytoplasmic compartments) and Bcl-2, whereas negative for Ki-67 and p53. Postoperative stay was unremarkable. The patient was discharged home in stable condition. Currently, the patient has completed adjuvant radiotherapy. There is no evidence of recurrence so far in 10 months after surgery.

## 3. Discussion

Salivary gland tumors represent 1% to 4% of all human neoplasias [1]. Pleomorphic adenoma is the most common tumor that affects the aforementioned glands [1–3]. Rarely PA undergoes malignant transformation into Carcinoma Ex-Pleomorphic Adenoma (CEPA) or carcinosarcoma. It is even more uncommon that PA metastasizes. Among salivary gland neoplasms, metastasizing pleomorphic adenoma (MPA) constitutes an extremely rare group of tumors [2, 3]. Although apparently benign, the MPA-associated mortality could be as high as 22% [2]. According to Nouraei and colleagues' review of case reports, the 5-year disease-specific and disease-free survivals of patients suffering from MPA are 58% and 50%, respectively [3].

Most of the patients (81%) with MPA have a history of at least 1 local recurrence of PA prior to the detection of distant metastasis. The vast majority (60%) of MPA patients present with symptoms. Among these MPA-symptomatic patients, head and neck mass encompasses the most common complaint, followed in descending order by lower back pain (16%), abdominal mass (8%), cranial nerve palsy (8%), nasal obstruction and anosmia (8%), dyspnea (8%), acute spinal cord compression (4%), pathological fractures (4%), and hip pain (4%). In only 17% of the patients the detection of MPA resulted from the medical evaluation of a local recurrent PA. Generally, PA recurrence occurs after 5.0 ± 4.9 years of primary PA presentation. At the present time, it is impossible to determine which of the locally recurrent PA has the potential to metastasize. Therefore, an investigation of a recurrent PA should include a search for distant metastases. Local PA lesions are identified by positron emission tomography, and by extrapolation, metastatic lesions could also be identified. Nouraei et al. have used positron emission tomography successfully to find and identify primary PA and MPA. However, it does not constitute a standard of care yet. PET/CT scan use is favored by Nouraei et al. opinion based on successful, but limited experience [3].

Local recurrence of PA is apparently related to incomplete resection of the disease during the first surgery. Primary PA enucleation should never be performed as it increases the rate of local recurrence. Pseudopod extensions in PA account for recurrences rate from 20% to 45% after simple enucleation [6]. Therefore, excision of the involved gland should be performed in primary PA lesions to diminish the chances of recurrence. Recurrence may constitute the first step in the dissemination and further metastasis of this tumor. Hitherto, excision of the involved gland and adjuvant radiotherapy should be done in recurrent cases to prevent possible distant spread [2].

Metastasectomy, when feasible, is the mainstay of MPA treatment [3]. It conferred a significant survival advantage over nonoperative treatment on log-rank analysis (*P* < 0.01) [3]. Chemotherapy (CTX) and primary radiotherapy (XRT) were not very effective in this disease according to Nouraei et al. [3]. However, other authors support adjuvant XRT to avoid missing hidden local recurrence that could eventually spread to distant sites [2]. Selective Neck Dissection (SND) and postoperative radiotherapy are indicated for regional lymph nodes metastasis [7].

The paradoxical nature of MPA, with its benign histological appearance and its metastatic spread capacity, intrigues clinicians and academic scholars. Several hypotheses have been formulated in order to explain how a histological benign tumor may produce metastasis, such as previous radiation of the primary PA or previous surgical intervention that should favor seeding and permeation of blood or lymphatic vessels by tumor cells, followed by metastatic spread [2]. However, the data in literature have shown that they are not absolute requisites for metastatic disease to occur [2, 4, 5].

Czader et al. reported a case of MPA in which the patient presented with a solitary kidney tumor that showed the histological features of a PA in the absence of a previous or concurrent salivary gland neoplasm or salivary gland surgery [4]. Thirteen months after the removal of the kidney tumor, the patient presented an aggressive parotid tumor, which pathological examination yielded a CEPA [4].

Fujimura and colleagues described a case of a primary left submandibular PA with no history of previous surgical intervention that presented concurrently with left occipital bone lesion, where CEPA was discovered. Submandibular lesion showed characteristic features of benign-mixed tumor with absent mitotic activity, but minimal cellular atypia and infiltrative cell growth that had penetrated the tumor capsule in several sites. On the other hand, left occipital bone-mixed tumor showed a carcinomatous component, bone osteolysis, hypervascularity, 19.5% of positive Ki-67 cells, and tumor invasion to intra- and extracranial structures including the left transverse sinus, consistent with CEPA. The submandibular tumor revealed 0.16% of positive Ki-67 cells, which are at the same level of their control comprised by typical mixed tumor of salivary glands that yielded 0.12%–0.31% (mean 0.22%) positive Ki-67 cells. Bone scintigrams and tumor scintigrams showed high uptake only in left occipital bone and left submandibular gland, ruling out the possibility of tumor primary site other than the left submandibular gland. They concluded that the primary PA in left submandibular gland was eventually metastasized through infiltration of the tumor capsule in route to the occipital bone, where this established MPA subsequently underwent malignant transformation into CEPA. Fujimura et al. agree that the best treatment for this condition is wide local excision of primary site and metastatic tumors [5].

These cases have challenged the classical theory which proposes that PA surgical manipulations seed tumor cells and allow them to permeate blood vessels through which they spread and metastasize. As a result of the aforementioned evidence, new theories have risen. Czader et al. propose that MPA and CEPA are different stages along a common biological pathway in malignant mixed-tumors spectrum. They hypothesize that metastasis capability of MPA more likely occurs secondary to accumulation of genetic mutations [4]. The case reports done by Czader et al. and Fujimura et al. show that MPA can potentially progress overtime to a more malignant phenotype [4, 5]. 

Current methods of histological diagnosis cannot differentiate MPA from a benign PA. Medical history of previous or concurrent primary PA is essential to diagnose MPA. Although PA and MPA possess a similar and benign histology, their natural history is quite different. MPA constitutes an aggressive malignant entity. In an attempt to elucidate the malignant behavior of MPA, we performed Ki-67, p53, p16, and bcl-2 immunohistochemistry staining of our case sample.

Monoclonal antibody Ki-67 immunoreactivity is a powerful tool in determining the aggressiveness of malignant neoplasms [8]. In our case, the tumor expression of Ki-67 was found negative. Luukkaa et al. showed that the extent of Ki-67 antigen expression in salivary gland cancer patients is inversely proportional to their survival. In the cases of low-grade tumors, the immunohistochemistry was mainly negative [8]. Katori et al. performed immunohistochemical analysis of Ki-67 index in both CEPA and PA, observing a significant increase in Ki-67 expression in patients who died of CEPA or who had CEPA residual disease as compared with patients with PA and those who were alive and without CEPA [9].

The p16 protein acts as a tumor suppressor leading to cell cycle arrest by blocking CDK4 and 6. Its inactivation or decreased expression has been reported in various human carcinomas [10]. In our case, we analyzed nucleus and cytoplasm p16 immunoreactivity, yielding similar positive expression in both cell compartments. Hu and colleagues discovered that malignant cell components of CEPA showed significantly lower p16 expression in the nuclei and significantly higher expression in the cytoplasm when compared to their benign components [10]. Though further studies need to be done, this could suggest that p16 overexpression in the cytoplasm along with decreased p16 expression in the nucleus may be important in the evolution of malignant transformation. A nuclear highly positive p16 could be then interpreted as belonging to tumor cells whose cell cycles are still being regulated. Nonetheless, no sufficient data is available to conclude that higher nuclear p16 positivity alone is enough to protect the cell from undergoing malignant transformation at some point in its life.

The p53 tumor suppressor gene mutation is the most common genetic alteration found in human cancers. This mutation also constitutes an early event in the malignant transformation of PA [11]. Our MPA case turned to be negative for p53 mutation. A recent Korean study showed that p53 is negative in benign PA [12]. Similarly, Alves et al. in Brazil found that mutant p53 is not generally expressed in PA [1]. Whether or not p53 mutation has value as a prognostic marker is unresolved; for instance, Luukkaa et al. reported that p53 immunohistochemistry did not correlate with clinical behavior nor overall survival in salivary gland cancer [8].

Bcl-2, deriving its name from B-cell lymphoma 2, is the first gene shown to be involved in the process of apoptosis. Bcl-2 expression in CEPA and PA is still undergoing study. Our sample resulted positive to Bcl-2 immunoreactivity. According to Yáñez et al., who analyzed a group of cases of benign PA, all of their samples were positively immunoreactive to Bcl-2 [13]. In a separate study, Aoki et al. also found 33 out of their 35 samples of PA positive for Bcl-2 [14]. On the other hand, Sunardhi-Widyaputra and Van Damme described strong Bcl-2 reactivity in the malignant areas of CEPA [15]. Thus, Bcl-2 participation in malignant transformation of PA needs further exploration.

Inconsistencies among authors may reflect small sample size and differences in experimental protocols, including the specificity and sensitivity of the antibodies used. Therefore, findings should always be interpreted with caution. Further studies that could include a larger sample of MPA cases as well as a wide variety of other benign PA and CEPA are needed to elucidate the intriguing nature, biological mechanisms, and genetic mutations that impart metastatic and malignant behavior to MPA, despite their benign histological appearance.
